# Photosynthesis in *Chromera velia* Represents a Simple System with High Efficiency

**DOI:** 10.1371/journal.pone.0047036

**Published:** 2012-10-10

**Authors:** Antonietta Quigg, Eva Kotabová, Jana Jarešová, Radek Kaňa, Jiří Šetlík, Barbora Šedivá, Ondřej Komárek, Ondřej Prášil

**Affiliations:** 1 Department of Marine Biology, Texas A&M University at Galveston, Galveston, Texas, United States of America; 2 Department of Oceanography, Texas A&M University, College Station, Texas, United States of America; 3 Institute of Microbiology, Academy of Sciences of the Czech Republic, Třeboň, Czech Republic; 4 Faculty of Sciences, University of South Bohemia, České Budějovice, Czech Republic; University of Connecticut, United States of America

## Abstract

*Chromera velia* (Alveolata) is a close relative to apicomplexan parasites with a functional photosynthetic plastid. Even though *C. velia* has a primitive complement of pigments (lacks chlorophyll *c*) and uses an ancient type II form of RuBISCO, we found that its photosynthesis is very efficient with the ability to acclimate to a wide range of irradiances. *C. velia* maintain similar maximal photosynthetic rates when grown under continual light-limited (low light) or light-saturated (high light) conditions. This flexible acclimation to continuous light is provided by an increase of the chlorophyll content and photosystem II connectivity under light limited conditions and by an increase in the content of protective carotenoids together with stimulation of effective non-photochemical quenching under high light. *C. velia* is able to significantly increase photosynthetic rates when grown under a light-dark cycle with sinusoidal changes in light intensity. Photosynthetic activities were nonlinearly related to light intensity, with maximum performance measured at mid-morning. *C. velia* efficiently acclimates to changing irradiance by stimulation of photorespiration and non-photochemical quenching, thus avoiding any measurable photoinhibition. We suggest that the very high CO_2_ assimilation rates under sinusoidal light regime are allowed by activation of the oxygen consuming process (possibly chlororespiration) that maintains high efficiency of RuBISCO (type II). Despite the overall simplicity of the *C. velia* photosynthetic system, it operates with great efficiency.

## Introduction

Most of all the diverse assemblage of eukaryotic oxygenic photosynthetic autotrophs present today belong to either the green (chlorophyll *b*-containing) or red (chlorophyll *c*-containing) plastid lineages [1], [2], [3]. There is however a subgroup of non-photosynthetic relatives, thought to have lost their plastids secondarily. The apicomplexans, which are non-photosynthetic sporozoan parasites (e.g., the malaria organism, *Plasmodium falciparum*), have a relic unpigmented plastid (apicoplast) indicating that the ancestors of these organisms were once photosynthetic, and that part of the plastid metabolic machinery is indispensable to the present organism. This may include the fatty acid synthesis enzymes [4] and isoprenoid biosynthesis [5]. Recently, two distinct families were described - *Chromeraceae* and *Vitrellaceae* [6] which include *Chromera velia* and *Vitrella brassicaformis* respectively. While apicomplexans are not currently placed in the polyphyletic group ‘algae’ by taxonomists, their algal roots have been long acknowledged [7], [8], [9].

*C. velia* (Chromerida, Alveolata) associated with the scleractinian coral *Leptastrea purpurea* was isolated in 2001 by Moore et al. [10] from Sydney Harbour (Australia). This is the first extant relative of apicomplexan parasites discovered to have a heritable functional photosynthetic plastid. *C. velia* plastid shares an origin with the apicoplasts, is surrounded by four membranes, pigmented with chlorophyll (chl) *a* and various carotenoids. Gene phylogenies relate the apicoplasts to the chloroplasts of peridinin-containing dinoflagellates [3], [11], [12]. Yet, *C. velia* plastids differ from those in dinoflagellates: they lack the accessory pigment chl *c* [10] and operate modified heterotrophic heme synthesis pathway [13]. It has been suggested that the ancestor of peridinin dinoflagellates and apicomplexans possessed a photosynthetic chromalveolate plastid containing chl *a* and *c* [7], [8]. Other dinoflagellates, in a series of complex events not discussed herein [see 7,11,12], now have fucoxanthin-containing plastids or green plastids, and up to 50% of others lost (and did not replace) their chromalveolate plastid resulting in heterotrophy [7], [8]. Nonetheless, many dinoflagellates today can still switch between autotrophy, mixotrophy and heterotrophy depending on environmental conditions. Zooxanthellae, a group of symbiotic dinoflagellates, have important relationships with corals and other invertebrates [14], [15], [16], [17]. Like many zooxanthellae, *C. velia* can live independently from its host and is culturable. With the discovery of *C. velia*, we now have a model organism to study apicomplexan evolution and zooxanthellae photosynthesis.

There has been a flurry of publications since the pivotal paper of Moore et al. [10] announcing the discovery of this unique phototroph. Keeling [9], Oborník et al. [18] and Janouškovec et al. [19] have re-evaluated and revised current views on plastid distribution in the red lineage with *C. velia* now providing an important “missing link”. Keeling [9] reported that the appearance of Chromera has, along with other cryptic plastids, provided important support for the chromalveolate hypothesis proposed by Cavalier-Smith some 10 years earlier (see 7) and more importantly, transformed the view of plastid distribution in the red lineage. Janouškovec et al. [19] conducted a careful phylogenetic analysis of plastid genomes to find extant plastids of apicomplexans and dinoflagellates were inherited by linear descent from a common red algal endosymbiont. It appears that plastids of heterokont algae and apicomplexa all derive from the same endosymbiosis. Okamoto and McFadden [20] concluded that the discovery of *C. velia* ends the debate on the origin of apicoplasts, providing strong evidence for origins with a red algal endosymbiont. Oborník et al. [18] instead focused on the pathway by which apicomplexa evolved from free-living heterotrophs through phototrophs to being the omnipresent obligatory intracellular parasite. More recently, Oborník et al. [6], [21] presented a careful examination of the morphology and ultra structure of multiple life cycle stages; Weatherby et al. [22] provide details of the cell surface and flagella morphology of the motile form of *C. velia*; Sutak et al. [23] provide details of a nonreductive iron uptake mechanism while Guo et al. [24] reported that both nutrient concentrations and salinity are important in regulating the transformation of immotitle-motile *C. velia*. Kořený at al. [13] found that unlike other eukaryotic phototrophs, *C. velia* synthesizes chl from glycine and succinyl-CoA, Kotabová et al. [25] found that fast de-epoxidation of violaxanthin in *C. velia* enables highly efficient non-photochemical fluorescence quenching (NPQ) and Pan et al. [26] published a detailed phylogenetic analysis of the light-harvesting antennae of *C. velia*. Recently, Botté et al. [27] identified plant-like galactolipids and Leblond et al. [28] determined sterols in a Chromera. Given the potential for *C. velia* in the screening of anti-apicoplast drugs for the treatment of malaria (*Plasmodium* sp.) and diseases caused by related parasites (e.g., *Toxoplasm*a), Okamoto and McFadden [20] concluded that “the little alga from the bottom of Sydney Harbour” may eventually be enlisted in developing new treatments for these diseases using herbicides which will attack the photosynthetic apparatus.

However, to date, there is no information on the properties and efficiency of photosynthesis in *C. velia* which displays simple pigmentation (only chl *a* together with violaxanthin, ß,ß-carotene and a novel isofucoxanthin-like carotenoid but without any accessory pigments like chl *c* [10]) and utilizes the primitive form (type II) of RuBisCO [19]. Herein, we investigated photosynthesis in *C. velia* in detail (electron transport and O_2_ evolution in Photosystem (PS) II, ^14^C fixation rates in Calvin-Benson cycle, pigment composition) together with its ability for photoacclimation to both continuous “low” and “high” light (15 and 200 µmol photons m^−2^ s^−1^) as well as its response to “natural” changes in irradiance provided with a sinusoidal light:dark regime. We found that photosynthesis in *C. velia* represents a simple system with surprisingly high efficiency. *C. velia* protects itself against photoinhibition at high irradiance by utilizing NPQ, energy spillover and photorespiration. At low irradiances *C. velia* maximizes its performance by reorganizing its antennae to ensure a constant light-dependent rate of photosynthesis across all growth environments.

## Methods

### Organism and culture conditions

**Figure 1:**
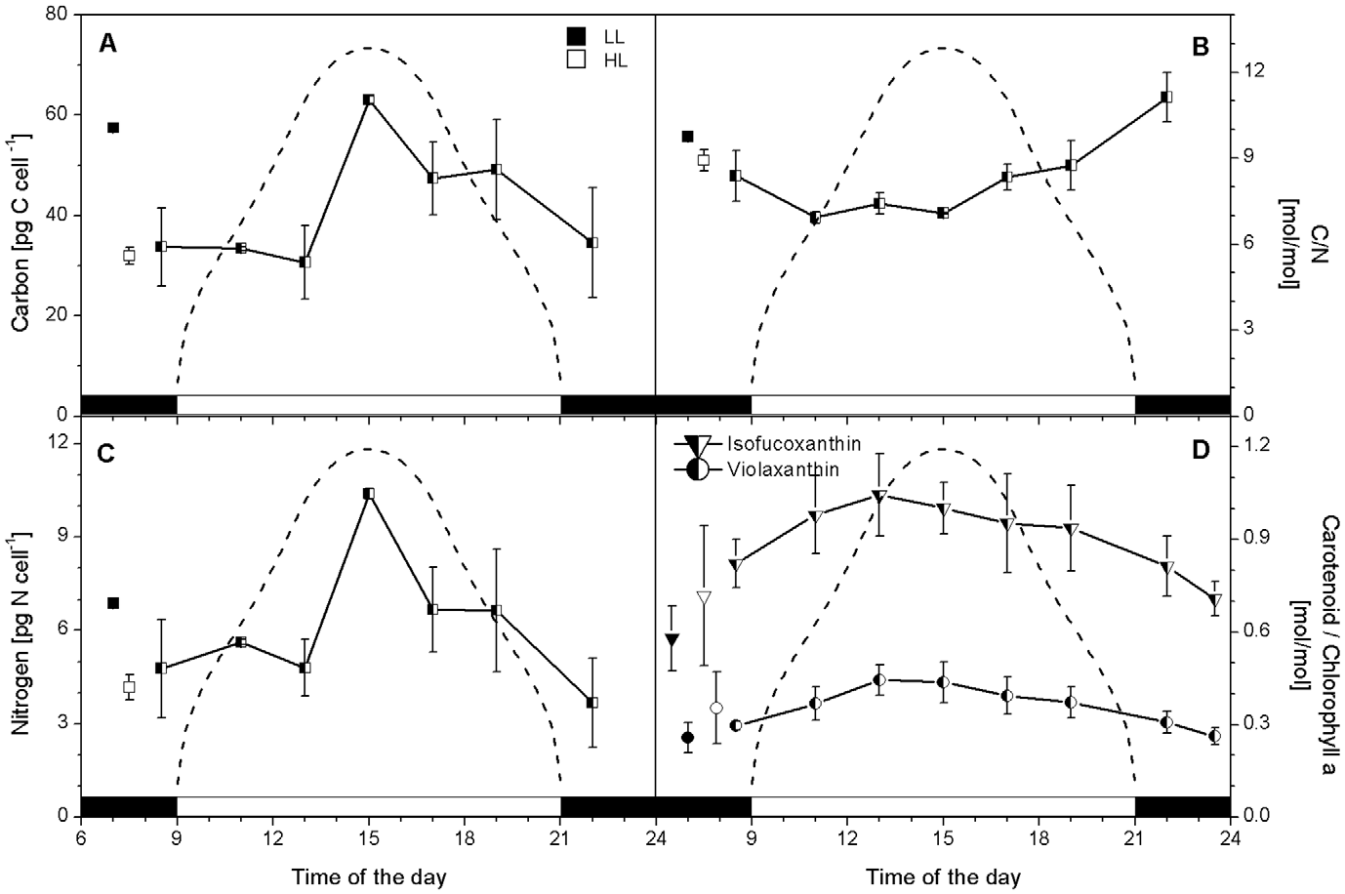
Changes in *C. velia* C, N, C∶N and carotenoids during a sinusoidal light∶dark cycle. Changes in C (pg C cell^−1^, panel A), the ratio of C∶N (mol∶mol, panel B), N quotas(pg N cell^−1^, panel C) and the major carotenoids relative to chl *a* (presented as ratio per chl *a*, panel D) are shown. Error bars are calculated as standard deviations of n≥3 replicated. Average values (plus error bars) measured for LL (▪) and HL (□) grown *C. velia* are included for comparison. The dashed line shows the light intensity during the light part of the cycle, with a midday peak of 500 µmol photons m^−2^ s^−1^.

*C. velia* (strain RM12) was maintained in f/2 culture medium and 28°C. For low light (LL) and high light (HL) experiments, cells were kept in semi-continuous batch growth with 24 h continuous light of 15 and 200 µmol photons m^−2^ s^−1^ respectively. For the sinusoidal light:dark cycle experiments, cells were grown under a 12∶12 h light∶dark cycle. Light intensity was controlled by computer [29] with a midday peak of 500 µmol photons m^−2^ s^−1^ (dashed curves e.g. in Fig. 1). Nutrient concentrations were saturating, pH buffered at 8.2, bubbling ensured CO_2_ supply and mixing. Cells were counted daily and size determined with a calibrated Coulter Counter (Beckman Mulitsizer III) equipped with a 70 µm aperture; cell densities were maintained between 1.0–2.0×10^6^ cells ml^−1^ by periodic dilutions with fresh f/2 medium. Specific growth rates (μ; day^−1^) were determined from μ = (ln c−ln c_0_)/t−t_0_) where c is the cell concentration and t is measured in days once cells had acclimated to the respective light treatment.

### Cell composition

Cultures (n≥3) were harvested onto precombusted (400°C, 4 hrs) GF/F filters and frozen until analysis of cellular carbon and nitrogen. Samples for pigment extractions were, in the same way as for fluorescence measurements, dark acclimated for 20 min prior to collection on GF/F filters and frozen immediately. Thawed filters were soaked in 100% methanol at −20°C and subsequently disrupted using a mechanical tissue grinder. Centrifugation (12000 g, 15 min) immediately before HPLC analysis on an Agilent 1200 chromatography system equipped with the DAD detector removed debris. Pigments were separated using a Phenomenex column (Luna 3μ C8, size 100×4.60 mm) at 35°C by applying a 0.028 M ammonium acetate/MeOH gradient (20/80) with a flow rate of 0.8 ml/min [30]. Eluted pigments were quantified by their absorption at 440 nm with consideration of their different extinction coefficients. For chl quantification, we used the same extract, but measured the sample on a UV/VIS spectrophotometer (Unicam UV 550, Thermo Spectronic, UK). Chl *a* concentration was calculated according to et al. [31].

### Fluorescence emission spectra

Room temperature fluorescence emission spectra were measured in cuvette with a SM-9000 spectrophotometer (Photon Systems Instruments, Czech Republic) for blue light excitation (λ = 464 nm) with a dark acclimated sample in the *F*_m_ (maximum fluorescence) state induced by a saturating pulse according to Kaňa et al. [32]. 77 K Chl fluorescence emission spectra were measured using the Aminco Bowman series 2 spectrofluorometer (Thermo Fisher Scientific, USA). The excitation was at 435 nm and 4 nm slit width. The emission spectra were recorded in 0.4 nm steps from 600 to 800 nm, with 1 nm slit width. The instrument function was corrected by dividing raw emission spectra by simultaneously recorded signal from the reference diode. Spectra were normalized to 690 nm. Fluorescence nomenclature is summarized in Table 1.

**Table 1:** Abbreviations, equations and units.

*α*	photosynthetic efficiency; measured from the initial slope of a PI curve	mgC mg chl *a* ^−1^ s^−1^/µmol photons m^−2^ s^−1^
a*_PSII_	chl a-specific PSII absorption coefficient	m^2^ [mg chl a]^−1^
Ag	gross photosynthetic rate	µmol O_2_ mg chl *a* ^−1^ h^−1^
*c*	cell concentration	cells mL^−1^
Chl *a*	chlorophyll *a*	pg cell^−1^
E_k_	index of light saturation = P_m_/α	µmol m^−2^ s^−1^
ETR_PSII_	Electron transport rate	µmol electrons mg chl *a* ^−1^ h^−1^
*F*_o_ and *F*_o′_	minimal fluorescence yield in the dark and in the light respectively	relative units
*F*_m_ and *F*_m′_	maximum fluorescence yield in the dark and in the light respectively	relative units
*F*_v_	variable fluorescence yield = *F*_m_ *- F*_o_	relative units
*F*_v_/*F*_m_	maximum quantum yield of photochemistry = (*F*_m_ *- F*_o_)/*F*_m_	relative units
*F_q_*	Difference between fluorescence yields = *F*_m_′ *- F*_t_	relative units
*F_t_*	actual fluorescence level at a given time excited by the actinic light	relative units
ΦPSII	effective quantum yield of PSII photochemistry (Genty's parameter) = (*F*_m′_-*F*_t_)/*F*_m′_	relative units
HL	high light	
LL	low light	
n	number of replicates performed for a given experiment	
NPQ	non-photochemical quenching = (*F*_m_-*F*_m′_)/*F*_m′_	relative units
*p*	Connectivity factor	
P^max^	maximum chl-specific carbon fixation rate	mgC mg chl *a* ^−1^ h^−1^
PS I	photosystem one	
PS II	photosystem two	
PQ	Photosynthetic quotient	
PTOX	plastid-localized terminal oxidase enzyme	
qP	photochemical quenching	relative units
RC_PSII_/Chl *a*	photosynthetic unit size	m^2^ (mol Chl *a*)^−1−1^
σ_PSII_	effective absorption cross section of PSII under dark acclimation	A^2^ PSII
t	time	day
μ	specific growth rate = (ln c - ln c_0_)/(t - t_0_)	day^−1^

### Variable fluorescence measurements

Chlorophyll fluorescence was measured using a double-modulation fluorometer FL-3000 (Photon System Instruments, Czech Republic). Before measurements started, cells were dark acclimated for 20 min to oxidize the electron transport chain. A multiple turnover saturating flash was applied to measure the maximum quantum yield of photochemistry of PS II (*F*_v_/*F*_m_) according to (*F*_m_-*F*_o_)/*F*_m_ where the difference between the maximum (*F*_m_) and minimal fluorescence (*F*_o_) is used to calculate the variable fluorescence (*F*_v_) [33]. Cells were then illuminated with an orange actinic light (625 nm; 480 µmol photons m^−2^ s^−1^). After 2 min, another saturating flash was applied and NPQ calculated as (*F*_m_-*F*_m_′)/*F*_m_′ in which case *F*_m_′ is the maximum fluorescence measured in the light. Photochemical quenching (qP) was calculated as (*F*_m_′-*F*_t_)/(*F*_m_′-*F*_o_′). The effective quantum yield of PSII photochemistry (Genty's parameter, Φ_PSII_) was calculated as (*F*_m_′-*F*_t_)/*F*_m_′, where *F*_t_ was the actual fluorescence level at given time excited by the actinic light.

Fast rate repetition fluorescence was measured using specially designed FM 3500 fluorometer (Photon Systems Instruments, Czech Republic). After 20 min dark acclimation, a series of 100 blue (463 nm) single turnover (1 µs) saturating flashes for sequential PSII closure were applied. This was done for eleven levels of blue (463 nm) actinic light intensities (0–1650 µmol photons m^−2^ s^−1^). The data were fitted to model of Kolber et al. [33] including parameters such as maximum and minimal fluorescence, effective PSII cross-section (σ*_PSII_*) and connectivity (*p*). These parameters were used for calculation of the electron transport rate *ETR_PSII_* as σ*_PSII_ (F*_q_ *′/F*_v_ *′)*/*(F*_v_/*F*_m_ *) E*, where *F*_q_ *′* is (*F_m_′-F′*) and *E* (light intensity) according to Suggett et al. [34]. The specific absorption of PSII (a*_PSII_) was calculated as (σ*_PSII_ (RC_PSII_/chl a*))/(*F*_v_ *′/F*_m_ *′*), where *RC_PSII_/chl a* is equal to 0.002 [34].

### Gas exchange measurements

Photosynthesis and dark respiration rates at 28°C were measured using a Clark-type oxygen electrode (Theta 90, Czech Republic) in the presence of 1 mM sodium bicarbonate. Light intensity in the electrode chamber was measured using a calibrated microspherical quantum sensor US-SQS/A (Walz, Germany) and light meter LI-250 (Li-Cor, USA). Gross photosynthesis (A_g_) was calculated from the slope of O_2_ evolution at a saturating irradiance plus the slope of respiratory O_2_ utilization measured in the dark after the light exposure. O_2_ evolution rates were normalized to chl *a*.

### ^14^C fixation

The relationship between photosynthesis and irradiance was determined using the small volume ^14^C incubation method of Lewis and Smith [35]. Cultures were kept in the dark for 20 mins before starting by spiking an aliquot (25 ml) of culture with ^14^C-sodium bicarbonate (MP Biochemicals, USA; final concentration of 1 µCi ml^−1^) and incubating for 40 mins at 28°C and a range of light intensities from 5 to 1500 µmol photons m^−2^ s^−1^. Triplicate samples for background counts (with 100 µL of buffered formalin) and total counts (with 250 µL of phenethylamine and 5 ml of Ecolume scintillation cocktail) were prepared at the start. Buffered formalin (100 µL) terminated the reactions; samples were acidified with 50% HCl (1 mL) were left overnight to purge off unincorporated label before disintegrations per minute were counted on a calibrated Tricarb 1500 Scintillation Counter. Dissolved inorganic carbon concentrations were determined in a cell-free medium by the Gran titration technique described by Butler [36]. Photosynthesis-irradiance curves were fitted using P = P_max_ × tanh (α×E/P_max_) according to Jassby and Platt [37] where the maximum chl-specific carbon fixation rate (P_max_ =  mg C mg chl^−1^ h^−1^) and the initial slope of the curve (α) = mg C mg chl^−1^ h^−1^ (µmole photons m^−2^ s^−1^)^−1^ were estimated from measurements of photosynthesis (P) and irradiance (E). The index of light saturation (E_k_; µmol m^−2^ s^−1^) was calculated as P_max_/α.

## Results

All findings are presented as averages of n≥3 cultures plus/minus standard deviations.

### Basic physiological parameters

Growth rates were determined once cultures of *C. velia* had acclimated to the respective irradiance (Table 2). While LL cells grew faster (0.21±0.02day^−1^) than those at HL (0.16±0.01 day^−1^), cells growing on the sinusoidal light∶dark cycle had the fastest overall growth rate of 0.37±0.01 day^−1^. Cell size was dependent on the light intensity for growth and light regime (Table 2), *C. velia* cell diameter decreased in the following order: LL (6.87±0.09 µm), sinusoidal cycle (6.07±0.63 µm) and HL (5.68±0.31 µm).

**Table 2:** Summary of cellular responses in *C. velia* grown under three different photon treatments.

	LL	HL	Sinusoidal light∶dark cycle	Response in sinusoidal cultures
Irradiance µmol photons m-2 s-1	15	200	Max. 500	Sinusoidal function
Irradiance regime	24 h continuous	24 h continuous	12 h∶12 h light∶dark	
Photon dose per day mol photons m-2 day-1	1.3	17.3	13.2	
Growth rate d-1	0.21±0.02	0.16±0.01	0.37±0.01	
Cell size µm	6.87±0.09	5.68±0.31	6.07±0.63	no change
C quota pg cell-1	57±1	32±2	53±12	See Fig. 1A
C∶N mol∶mol	9.8±0.1	8.9±0.4	8.4±0.7	See Fig. 1B
N quota pg cell-1	6.88±0.14	4.18±0.40	6.4±1.7	See Fig. 1C
Chl a pg cell-1	0.60±0.08	0.21±0.05	0.45±0.04	no change
violaxanthin/Chl *a* mol∶mol	0.26±0.05	0.35±0.12	0.36±0.07	See Fig. 1D
isofucoxanthin/Chl *a* mol∶mol	0.58±0.11	0.72±0.23	0.91±0.11	See Fig. 1D
ß,ß-carotene/Chl *a* mol∶mol	0.030±0.006	0.034±0.009	0.045±0.007	no change
total carotenoids/Chl *a* mol∶mol	0.87±0.15	1.11±0.31	1.31±0.18	See Fig. 1D
Chl *a* specific absorption of PSII a*PSII	0.0071±0.0002	0.0101±0.0003	0.0109±0.0003	Not shown

Cellular C and N concentrations for *C. velia* were dependent of the irradiance for growth as well as the time of day (Table 2; Fig. 1A, B, C). The average cellular C quota was significantly higher in LL (57 pg C cell^−1^±1) than in HL (32 pg C cell^−1^±2) (*p* = 0.002; n = 2) cells but this was not the case for N quotas (6.88 pg N cell^−1^±0.14 and 4.18 pg N cell^−1^±0.40 respectively, *p* = 0.093; n = 2). However, given the different cell sizes, the average cellular densities of C and N were almost identical for both treatments (0.33–0.34 pg C µm^−3^ and 0.041–0.044 pg N µm^−3^, respectively). In the sinusoidal grown *C. velia* (Fig. 1 A, C), C quotas increased throughout the light period from predawn values of 34 pg C cell^−1^ (±8) to 63 pg C cell^−1^(±1) in the middle of the day corresponding to a 85% increase (paralleled by a 116% increase in N quotas). Within an hour of lights off, C and N quotas were back down to predawn levels. The average cellular density of C was comparable to LL and HL cultures (0.36 pg C µm^−3^) but the cellular density of N was higher (0.052 pg N µm^−3^) in the sinusoidal cultures. Molar C∶N ratios changed throughout the light photoperiod (Fig. 1B).

### Pigment composition

Chl *a* concentrations responded to the irradiance for growth (Table 2). In *C. velia*, cellular chl *a* concentrations were three times higher in LL cells compared to those growing at HL (0.60 ± 0.08 pg chl *a* cell^−1^ and 0.21 ± 0.05 pg chl *a* cell^−1^respectively). Chl *a* concentrations did not vary significantly (*p* = 0.236; n = 21) throughout the sinusoidal light∶dark cycle (0.45± 0.04 chl *a* pg cell^−1^). Despite growing at very different irradiances, the cellular density of pigments was comparable for LL (3.5 fg chl *a* µm^−3^) and sinusoidal grown *C. velia* (3.8 fg chl *a* µm^−3^) but were the lowest when cells were grown at HL (2.2 fg chl *a* µm^−3^).

Unlike its closest extant photosynthetic relatives, *C. velia* lacks chl *c* and only expresses a limited set of carotenoids [10]. Changes in the fraction of violaxanthin, isofucoxanthin, ß,ß-carotene and total carotenoids were examined relative to chl *a* (Table 2). In HL relative to LL grown cells, there was in total, 28% more carotenoids (1.11 ± 0.31 and 0.87 ± 0.15 respectively), specifically 35% more violaxanthin (0.35 ±0.12 versus 0.26±0.05 respectively) and 24% more isofucoxanthin (0.72±0.23 versus 0.58±0.11 respectively). ß,ß-carotene was a minor component of the accessory pigment pool, and did not change significantly between HL and LL grown *C. velia* (0.034±0.009 and 0.030±0.006 respectively; *p* = 0.237; n = 3). In *C. velia* cells growing on a sinusoidal regime, predawn total carotenoids: chl *a* ratios were similar to those of HL grown cells (1.17 ± 0.09), increasing to 1.55 (± 0.17) before noon, then decreasing to 1.00 (± 0.07) several hours post illumination (Fig. 1D). ß,ß-carotene also changed significantly (*p*<0.001; n = 23) throughout the sinusoidal cycle (not shown), following a similar trend to total carotenoids. At midday, there was 47% more violaxanthin and 22% more isofucoxanthin present in *C. velia* than before dawn. Ratios of accessory pigments to chl *a* at midday were similar, albeit higher, than those measured in HL growing *C. velia*. When translated to cellular densities, then the density of violaxanthin was similar in LL and HL cells (1.22 and 1.15 fg µm^−3^, respectively, while it almost doubled in the sinusoidal cells (2.54 fg µm^−3^). The cellular density of light harvesting isofucoxanthin was also the highest in the sinusoidal grown cells (5.8 fg µm^−3^), than in LL cells (3.08 fg µm^−3^) and HL cells (2.36 fg µm^−3^).

### Rearrangement of light harvesting complexes

**Figure 2:**
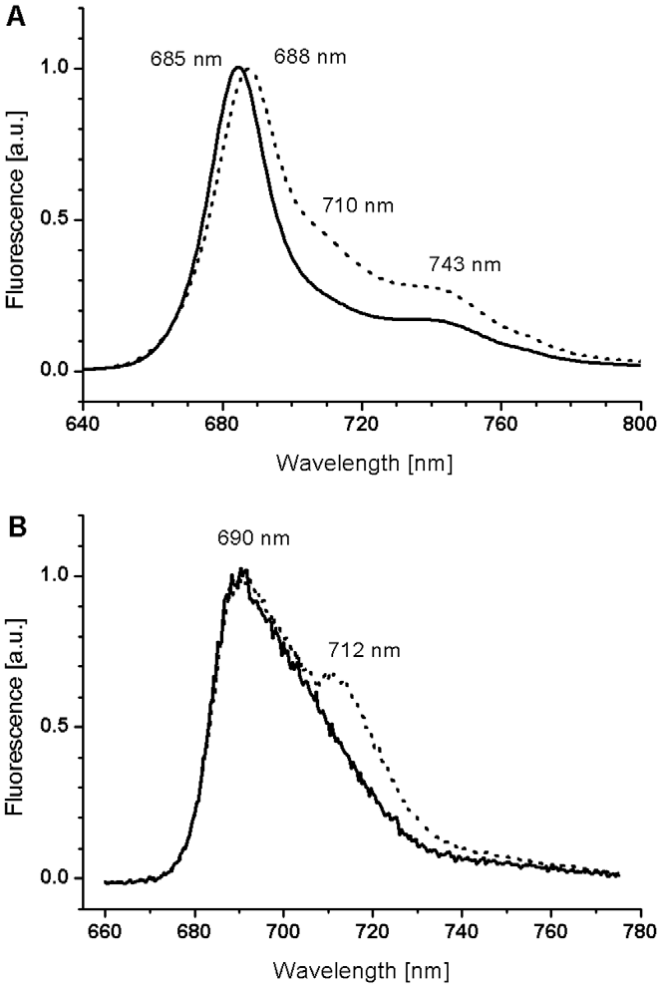
Room temperature and 77K fluorescence emission spectra for *C. velia* grown under LL and HL. Spectra were normalized at the chlorophyll a fluorescence emission maxima. Given the fluorescence spectra was identical for HL and sinusoidal light∶dark cycle grown *C. velia*, we only show the results for the HL grown cells.

The changes in arrangement of light harvesting complexes during acclimation to HL and LL were deduced from fluorescence emission spectroscopy. Spectra were measured at room and at low (77 K) temperatures. The room temperature (RT) fluorescence emission spectra of *C. velia* detected a clear red-shift of the PSII maximum between HL and LL grown *C. velia* (from 685 nm for HL to 688 nm for LL, Fig. 2A). As changes in intensities of both emission bands observed at RT (685 nm and 688 nm) showed similar kinetics during fluorescence induction with continuous light (data not shown), we attribute them to the fluorescence emission of PSII core proteins. In LL grown *C. velia*, an additional red-shifted fluorescence band at 710 nm can be seen at RT (Fig. 2A). Since PSI is not known to emit at RT and the intensity of the 710 nm fluorescence band at RT showed similar variability as the PSII emission band at 685 nm (data not shown), this indicates its origin in some red-shifted antennae of PSII.

At 77 K, a major emission band with maximum at 690 nm was observed (Fig. 2B). Under LL conditions, an additional red-shifted emission maximum at 77 K was observed as a shoulder at 712 nm (Fig. 2B). These results indicate that acclimation to different light intensities induces antennae reorganization. Since there is no distinct band that can be attributed to PSI fluorescence in the 77 K emission spectra, we cannot determine whether light acclimation also affected the stoichiometry of PSI and II. The emission spectra of cells during the sinusoidal light∶dark cycle in *C. velia* were identical to HL grown *C. velia* (not shown).

### Variable fluorescence parameters

**Figure 3:**
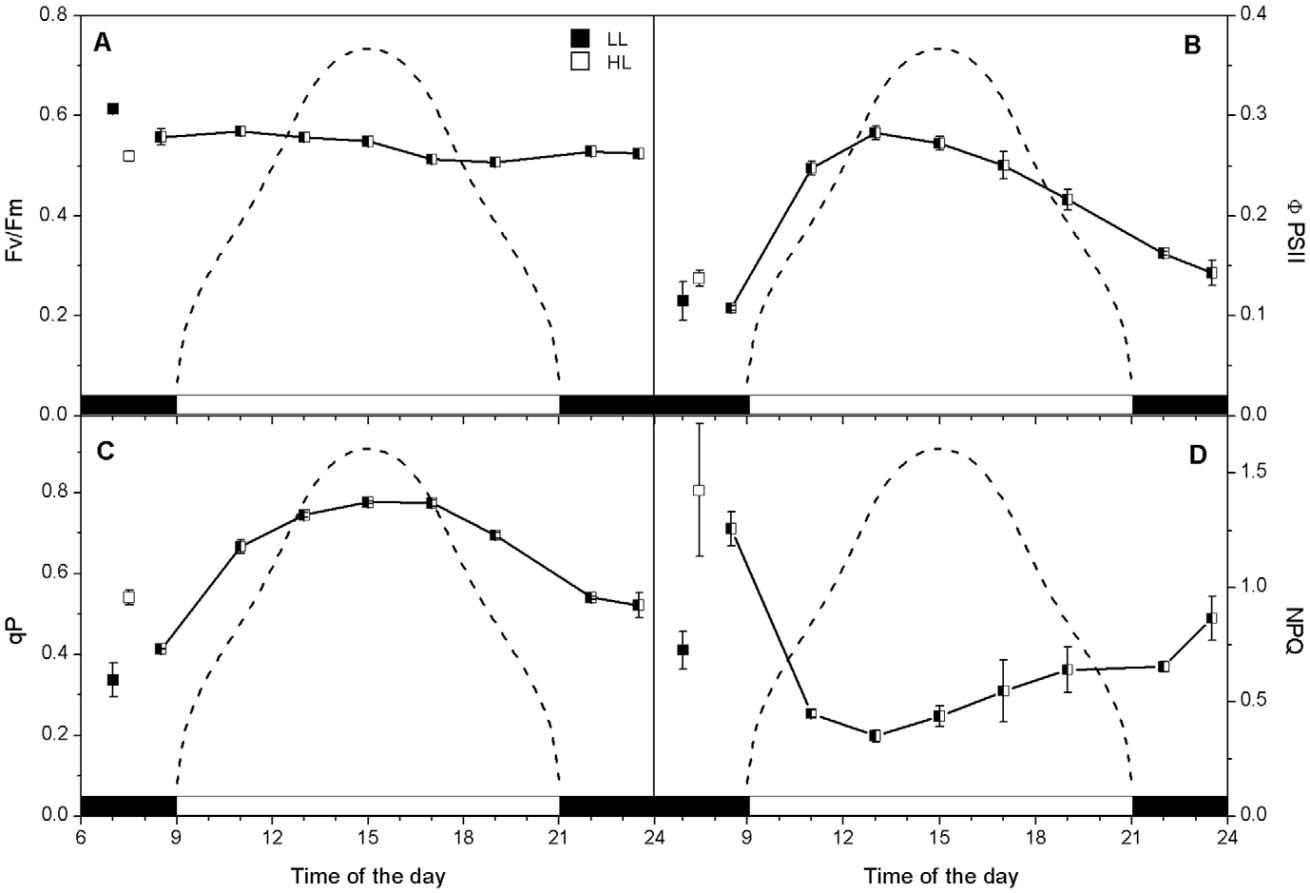
Changes in *C. velia* major fluorescence parameters during a sinusoidal light∶dark cycle. Changes in the *F*_v_/*F*_m_ (panel A), Φ_PSII_ (panel B), qP (panel C) and NPQ (panel D) are shown. Error bars are calculated as standard deviations of n≥3 replicated. Average values (plus error bars) measured for LL (▪) and HL (□) grown *C. velia* are included for comparison. The dashed line shows the light intensity during the light part of the cycle, with a midday peak of 500 µmol photons m^−2^ s^−1^.

The maximal efficiency of PSII photochemistry, *F*_v_/*F*_m_, was 0.61 (±0.01) for LL grown *C. velia* (Table 3). By contrast, HL grown *C. velia* had an *F*_v_/*F*_m_ ratio of 0.52 (±0.01) (Table 3) indicating a decrease in the maximal efficiency of PSII photochemistry. Similarly, during the sinusoidal cycle, *F*_v_/*F*_m_ ratios declined from 0.56 (±0.01) at the beginning of the light period to 0.51 (±0.01) by the evening and then started to increase again after the light was turned-off (Fig. 3A).

**Table 3:** Summary of physiological responses measured using fluorescence techniques in *C. velia* grown under three different photon treatments.

	LL	HL	Sinusoidal light∶dark cycle	Response in sinusoidal cultures
*F*_v_/*F*_m_	0.61±0.01	0.52±0.01	0.54±0.02	Fig. 3A
Φ_PSII_	0.11±0.02	0.14±0.01	0.21±0.06	Fig. 3B
qP	0.34±0.04	0.54±0.02	0.64±0.13	Fig. 3C
NPQ	0.73±0.08	1.42±0.29	0.65±0.29	Fig. 3D
1-qP	0.66±0.04	0.46±0.02	0.36±0.13	Not shown
σ_PSII_	307±8	380±6	444±8	Not shown
*p*	0.37±0.01	0.22±0.01	0.26±0.04	Not shown

(Note: *p* in this table refers to the connectivity factor).

The efficiency of PSII photochemistry in the light, Φ_PSII_ (also known as the Genty parameter), was slightly greater when *C. velia* was grown at HL (0.14±0.01) than at LL (0.11±0.02) (Table 3). In *C. velia* cells grown under a sinusoidal light∶dark cycle, the predawn value of Φ_PSII_ was similar to LL grown cultures (0.11±0.01), then increasing rapidly after onset of light and reaching the highest values before midday (0.28±0.01). Subsequently Φ_PSII_ started to decline towards the dark period to value of 0.14 (±0.01) (Fig. 3B).

qP reflects the number of open PSII reaction centers and denotes the proportion of excitation energy trapped by them – therefore the higher qP, the more efficient it is in utilization of incident light. We have found that HL relative to LL grown *C. velia* had higher qP values (0.54±0.02 and 0.34±0.04 respectively) (Table 3). In the sinusoidal *C. velia* cultures, we observed a gradual increase in qP with increasing light intensity during light period (Fig. 3C). Average predawn qP values were 0.41 (±0.01), while those at noon were almost double (0.78±0.01). qP started to decline already pre dusk to 0.52 (±0.03) after the onset of night, (Fig. 3C). All these results showed ramping up of the photosynthetic apparatus during mid-morning (qP increase, Fig. 3C) and the ability of *C. velia* to use most of the incident light for photosynthesis during maximal irradiance (no decay of qP at noon, Fig. 3C).

Given that NPQ is proportional to heat-dissipation of excitation energy in the antenna system in the dark acclimated state [see 25], or more simply, the amount of energy not used in photochemistry, changes in NPQ in the sinusoidal cultures reflect the cells dynamically responding to changes in irradiance for growth. NPQ was highest predawn (1.26±0.07) and declined rapidly after onset of light to a minimum 0.35(±0.03) before midday. From midday NPQ showed a continual increase (Fig. 3D) through to the dark period. NPQ was higher (in fact doubled) in HL grown *C. velia* (1.42±0.29) than LL cultures (0.73±0.08) (Table 3). These data show that *C. velia* can cope efficiently with increasing light intensity during diel cycle as NPQ values were minimal and relatively stable during the first half of light period (up to 15 h, see Fig. 3D), and the non-radiative energy dissipation was stimulated only afterwards.

### O_2_ evolution,^14^C fixation and the Photosynthetic quotient

**Figure 4:**
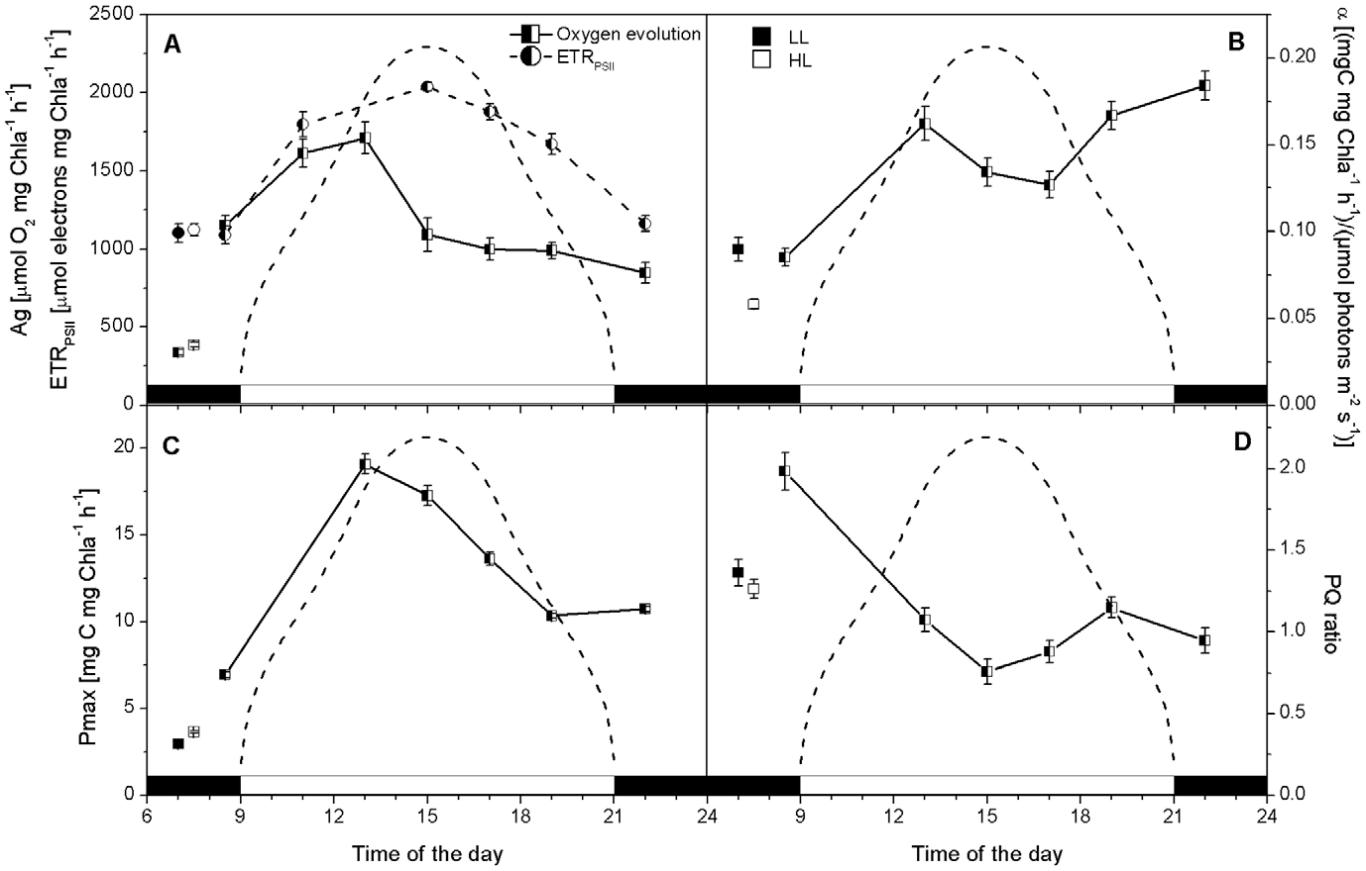
Changes in *C. velia* light and dark reactions during a sinusoidal light∶dark cycle. Changes in O_2_ evolution and ETR_PSII_ (panel A), alpha (photosynthetic efficiency, panel B), C fixation rates (panel C) and the photosynthetic quotient (PQ, panel D) are shown. Error bars are calculated as standard deviations of n≥2 replicated. Average values (plus error bars) measured for LL (▪) and HL (□) grown *C. velia* are included for comparison. The dashed line shows the light intensity during the light part of the cycle, with a midday peak of 500 µmol photons m^−2^ s^−1^.

The ability of *C. velia* to efficiently acclimate photosynthesis to wide range of constant irradiance was reflected in the values of the gross rate of O_2_ evolution. Ag was comparable for HL and LL *C. velia* when expressed on a per chl *a* basis (Table 4; Fig. 4A). This was also the case for ^14^C fixation, found to be 3.67±0.07 and 2.97±0.07 mg C mg chl^−1^ h^−1^for HL and LL *C. velia* respectively (Table 4; Fig. 4C).

**Table 4:** Summary of physiological responses measured by oxygen evolution and ^14^C fixation in *C. velia* grown under three different photon treatments.

	LL	HL	Sinusoidal light∶dark cycle	Response in sinusoidal cultures
A_g_ µmol O_2_ mg chl*a* ^−1^ h^−1^	338±19	386±16	1200±329	Fig. 4A
P_max_ µmolC mg chl*a* ^−1^ h^−1^	248±5	306±6	1084±381	Not shown
P_max_ mgC mg chl*a* ^−1^ h^−1^	2.97±0.07	3.67±0.07	13.0±4.6	Fig. 4C
ETR_PSII_ µmole electrons mg chl *a* ^−1^ h^−1^	1102±59	1120±38	1605±391	Fig. 4A
PQ	1.36±0.08	1.26±0.06	1.13±0.44	Fig. 4D
α mgC mg chl*a* ^−1^ s^−1^/µmol photons m^−2^ s^−1^	0.09±0.01	0.06±0.00	0.14±0.04	Fig. 4B
E_k_ µmol photons m^−2^ s^−1^	33±3	63±3	90±33	Not shown

We observed a pronounced ‘hysteresis effect’ in photosynthetic parameters of *C. velia* grown under the sinusoidal light∶dark cycle. The hysteresis effect represents an asymmetric response of photosynthesis to the same irradiance in the morning versus the afternoon [38], [39], [40] and references therein. We observed maximum O_2_ evolution rates before noon with reduced O_2_ evolution in the afternoon at the same light intensity (Fig. 4A). In the same way, the maximum ^14^C fixation rate was measured before noon was greater than that measured after (Fig. 4C). Thus, both dark and light photosynthetic reactions show the mid-morning maximum. However, while ^14^C fixation rates gradually decreased after the mid-morning peak, O_2_ evolution rates declined rapidly to ca. 60% at midday and then remained constant until late evening (Fig. 4A, C).

Changes in photosynthetic efficiency did not follow those for the maximum photosynthetic rate in the sinusoidal cultures (Fig. 4B); α increased steadily throughout the day to ca. 0.18±0.01 mgC mg chl^−1^ h^−1^ (µmole photons m^−2^ s^−1^)^−1^ with a midday depression. In both HL and LL grown *C. velia*, α values were similar to predawn values measured of the sinusoidal cycle (Fig. 4B), (0.06±0.00 and 0.09±0.01 mgC mg chl^−1^ h^−1^ (µmole photons m^−2^ s^−1^)^−1^ respectively).

We found the saturation intensity for carbon fixation (E_k_) was higher, in fact doubled, in HL grown relative to LL grown *C. velia* (63±3 µmole photons m^−2^ s^−1^ and 33±3 µmole photons m^−2^ s^−1^ respectively) (Table 4) but still below the growth irradiance for HL cultures. In sinusoidal cells of *C. velia*, E_k_ was much higher than in cultures growing under continuous light and followed the diel changes in irradiance, that is, E_k_ increased from the predawn value of 82±5 µmole photons m^−2^ s^−1^ to 129±9 µmole photons m^−2^ s^−1^ at midday and dropped back down to 58±3 µmole photons m^−2^ s^−1^ after the period of light (not shown). This indicates that it is possible for C. *velia* to attain a high E_k_, but not in the HL cells grown on continuous light.

The photosynthetic quotient (PQ) is defined by the molar ratio of the rate of oxygen production relative to carbon dioxide assimilated. We found the PQ ratio to be ∼1.3 when examining *C. velia* grown in continuous light, both in the HL and LL grown cultures (Table 4). *C. velia* cells growing on the sinusoidal light∶dark cycle modulated their photosynthetic quotient in response to the changing irradiance during the light period; with the midday minimum 0.76. While the photosynthetic quotient was still close to 1 an hour after dark, the predawn value was doubled (PQ≈2) (Fig. 4D).

## Discussion

We have measured the photosynthetic activities and photoacclimation strategies of the coral associated alveolate alga *Chromera velia*, the closest photosynthetic relative to apicomplexan parasites and dinoflagellate algae [10], [19]. The majority of reef building scleractinian corals contain endosymbiotic dinoflagellate algae (zooxanthellae) of the genus *Symbiodinium* [14], [15], [16], [17]. The photosynthetic performance of zooxanthellae is affected by environmental factors including ambient light (quantity and quality), temperature, CO_2_, and nutrient availability. The response to light is arguably the most important factor controlling productivity, physiology and ecology of corals [16], [40], [41], [42], [43], [44], [45]. Many studies have investigated photoacclimation strategies in scleractinian and other corals e.g., [14], [15], [16], [17], [43], [44], [45], [46], [47], [48] but studies of the free-living zooxanthellae are less common e.g., [14], [45], [50]. Studies have shown that corals and their symbiotic algae may be vulnerable to very high irradiances, expressed in the natural environment as a localized solar bleaching response (e.g., reduction in numbers of symbiotic dinoflagellates, loss of photosynthetic pigments, photoinhibition of photosynthesis, or a combination of these) [45], [48], [49], [51], [52].

### LL and HL acclimation strategies

To understand the basic physiological behavior and acclimation strategies we cultivated *C. velia* at continuous irradiance under two extreme light intensities: low (15 µmol m^−2^ s^−1^) and high (200 µmol m^−2^ s^−1^). LL *C. velia* had greater C, N and chl *a* quotas, higher *F*_v_/*F*_m_ in comparison to HL grown cells which in turn had more photoprotective carotenoids. The LL grown *C. velia* accumulated both C and N reserves and increased their light harvesting potential (package effect aside) relative to those grown at HL (Table 2). The acclimation to LL conditions also required substantial antennae reorganization, as indicated by appearance of an extra emission band (710 nm at RT, 712 nm at 77 K; see Fig. 2). Moreover, PSII reactions centers of LL grown *C. velia* are probably better interconnected as deduced from the 78% higher value of the connectivity parameter compared to HL grown cells (Table 3). On the other hand, there is probably also a redistribution of some PSII antennae towards PSI in LL grown cells. This is also observed in the reduction of chl *a* specific absorption of PSII by 29% (a*_PSII_; see Table 2) as well as the functional absorption cross section of PSII by 19% (σ_PSII_; see Table 3) in spite of the three times higher chl *a* concentrations. We suggest that the PSI abundance increases PSI activity that could be used for optimization of photosynthesis under light limited conditions.

The acclimation to HL conditions considerably decreased the chl content and increased carotenoid:Chl *a* ratio (Table 2). It was recently shown that both major carotenoids, isofucoxanthin as well as violaxanthin, contribute to light-harvesting in *C. velia* [25]. The increase in pigment content was much more pronounced for violaxanthin (35%) than for isofucoxanthin (24%); this is related to their photoprotective roles [25]. Violaxanthin in *C. velia* undergoes very fast de-epoxidation to zeaxanthin under the excessive irradiance that enables efficient photoprotection by NPQ [25]. In line with that, HL grown cells, containing appreciably more violaxanthin had NPQ values twice those measured in LL cells (Table 3). The NPQ reflects non-photochemical energy dissipation and reduces the excitation pressure over PSII in HL grown *C. velia* (see 1-qP lower by 30% in comparison with LL in Table 3). This reveals the ability of *C. velia* to protect itself under high-light growth conditions (200 µmol m^2^ s^−1^). As a result, in spite of the reduction of photosynthetic efficiencies (see the lower of *F*_v_/*F*_m_ and α in Tables 3 and 4), *C. velia* can maintain the rates of photosynthesis for both, light (O_2_ evolution, A_g_) and dark (C fixation, P_max_) reactions during growth under HL conditions (see Table 4).

Growth rates, C∶N ratios, Chl *a* and photosynthetic rates for *C. velia* are similar to those reported for *Symbiodinium* spp. e.g. [14], [45], [47] and other eukaryotic algae grown under continuous light e.g. [38], [53]. The low values of Φ_PSII_ measured in *C. velia* are similar to those measured in coral residing in the shallowest habitats [54]. This is usually interpreted as suggesting low efficiency of PSII but that cannot be the case in *C. velia* as we measured comparatively high rates of oxygen evolution. Alternatively, it can indicate redistribution (spillover) of excitation between PSII and PSI. Other experimental data (fluorescence recovery after photobleaching, R.Kaňa, unpublished) also suggest high mobility of *C. velia* antennae and thus support the spillover hypothesis. In the case of spillover the *F*_m_ fluorescence of PSII could be lowered by non-fluorescent PSI. Such limitation of excitation energy transfer to PSII at high irradiances allows *C. velia* to limit photodamage to the D1 protein in PSII. It seems that *C. velia* possess a unique organization of the antennae system where absorbed light is distributed among both photosystems to increase their efficiency.

### Photosynthesis under sinusoidal light∶dark cycle

The efficient photosynthesis in *C. velia* was further stimulated under sinusoidal light∶dark cycle that better simulates physiological conditions in nature. In this case, we measured very high rates of O_2_ evolution (up to 1708 µmol O_2_ mg chl^−1^ h^−1^) and ^14^C fixation (up to 19 mg C mg chl^−1^ h^−1^) during the light phase of the sinusoidal light∶dark cycle, some 4–5 times higher than those measured in cultures receiving continuous light. In addition, the sinusoidal light regime allowed greater flexibility and dynamics of the photosynthesis apparatus with more than 60% of NPQ (depending the time of day) recovered within 3 minutes of sampling, while only 40% recovered in cultures grown under continuous irradiance (data not shown). Hence, the diel periodicity in irradiance during the growth cycle is crucial to obtaining maximal photosynthetic rates (P_max_, A_g_) and consistent with the need of *C. velia* to maintain energetic balance within the primary photosynthetic reactions.

We also found that while the actual PSII photochemistry in the light (Φ_PSII_, Fig. 3B) tracked changes in ^14^C fixation and O_2_ evolution rates (mid-morning maximum and afternoon depression, see Fig. 4A, C), the maximal quantum yield of PSII (*F*_v_/*F*_m_) and qP were maintained until the late afternoon (Fig. 3A, C). This afternoon difference between the maximal capacity of PSII photochemistry (represented by *F*_v_/*F*_m_, Fig. 3A) together with qP (Fig. 3C) and actual efficiency of PSII in light (Φ_PSII_, Fig. 3B) correlated with gradual increase of NPQ during the afternoon (Fig. 3D). Stimulation of NPQ (Fig. 3D) could be the consequence of slower CO_2_ assimilation during the afternoon (Fig. 4C) resulting in slower ATP regeneration that would cause higher lumen acidification [55], a main stimulus of NPQ increase in *C. velia* [25]. Our results therefore suggest that optimization of light reactions during day period proceeds on the level of regulation of light-harvesting antennae (by NPQ, see Fig. 3 D), rather than through photoinhibitory destruction of core proteins of PSII. Further support for this comes from the lack of a significant mid-day depression in *F*_v_/*F*_m_, a sign of photoinhibition [56], [57]. Also, the excitation pressure over PSII (1-qP) during the highest irradiances was minimal (around 0.22); this excludes an overexcitation of PSII and therefore photoinhibition.

Hysteresis effects were observed during the sinusoidal light cycle when examining O_2_ evolution rates and ^14^C fixation rates (Fig. 4A, C). Indeed, *C. velia* appeared to be taking an ‘afternoon nap’. What we found interesting is that the afternoon depression varied between the light and dark reactions of photosynthesis. While ^14^C fixation rates were gradually decreasing after the mid-morning peak (Fig. 4C), O_2_ evolution rates declined rapidly to ca. 60% at midday and then remained constant until e in the evening (Fig. 4A). Given the culture conditions, the hysteresis we observed were not related to changes in nutrient and CO_2_ availability. Additional support for this comes from the C and N quotas which were not that different from Redfield (Fig. 1) and pH values which were close to 8.2 (not shown) throughout the sinusoidal light∶dark cycle. The decoupling between O_2_ evolution and ^14^C fixation rates during the midday and early afternoon maybe related to changes in photorespiration, the process whereby phototrophes fix O_2_ and liberate CO_2_ [58]. Photorespiration has been previously reported in zooxanthellae [see 16,59], but rates were generally found to be low, presumably because the type II RuBisCO was found exclusively in the pyrenoid [60] thus limiting exposure to O_2_ and/or due to the presence of a carbon concentrating mechanism [61].

Burris [62] reported that photosynthetic quotients could be even as low as 0.1 when photorespiration is the dominant process while little or no photorespiration takes place when PQs are > than 1. As we measured PQ's of 0.76 at midday (Fig. 4D), we suggest the presence of photorespiration in *C. velia*, but only with rather low rates, similarly as was observed for zooxanthellae. This would imply that *C. velia* switches from primarily production of glycolate and its excretion (the greater the glycolate, the lower the quotient) during the day to glycerol synthesis at night (PQ≈2, see Fig. 4D) [57]. The presence of photorespiration in *C. velia* suggests that it has a similar lifestyle to symbiotic dinoflagellates. The released carbon compounds (referred to as ‘junk food’ by Falkowski et al. [41]) are used by the coral for respiratory energy generation via the synthesis of energy-rich storage products such as lipids and starch [16], [41]. The high carbon fixation rates in *C. velia* may be associated with supplying carbon to this cycle.

Photorespiration is thought to be an evolutionary relic as primitive photosynthesis originated from the early atmosphere with very little oxygen and thus the early RuBisCO lacked discrimination between O_2_ and CO_2_ [58]. *C. velia* possess the primitive form of RuBisCO (type II) homologous to that of dinoflagellates that was acquired during evolution by the horizontal gene transfer from a proteobacterium [19]. The type II RuBisCO comprises only large subunits, has a high K_c_ but has a poor affinity for CO_2_ and discriminates CO_2_ from O_2_ less well than type I RuBisCO [58], [61], [63], [64]. We suggest that type II RuBisCO could be a reason for such a high ^14^C fixation rates observed in *C. velia*. However, type II RuBisCO is very sensitive to presence of O_2_ that could be used for its oxygenase activity and thus reduce carboxylation activity. Therefore, there must be a mechanism to reduce O_2_ accessibility to RuBisCO. One of the most known mechanisms that operates to suppress the oxygenase activity of RuBisCO is the carbon-concentrating mechanism (CCM) that accumulates C_i_ and elevates [CO_2_] around RuBisCO. *Symbiodinum* spp. fix inorganic carbon efficiently because they use CCMs [59], [61]. Nonetheless, even in zooxanthellae, CCM activity has not been found to be as high as might be expected [61]. Another possibility is the presence of special organelles (pyrenoids) that elevate [CO_2_] within chloroplasts. Pyrenoids are often present in dinoflagellates [65]; their formation appears to be correlated with CO_2_ fixation rates in *Gonyaulax* sp. [60] which also utilizes type II RuBisCO. Currently, we lack direct evidence of CCMs and pyrenoids in *C. velia* but given the very high carbon fixation rates parallel the highest O_2_ evolution rates mid-morning, it appears that *C. velia* is somehow able to build a sufficient carbon pool around RuBisCO so that it functions predominately as a carboxylase.

**Figure 5:**
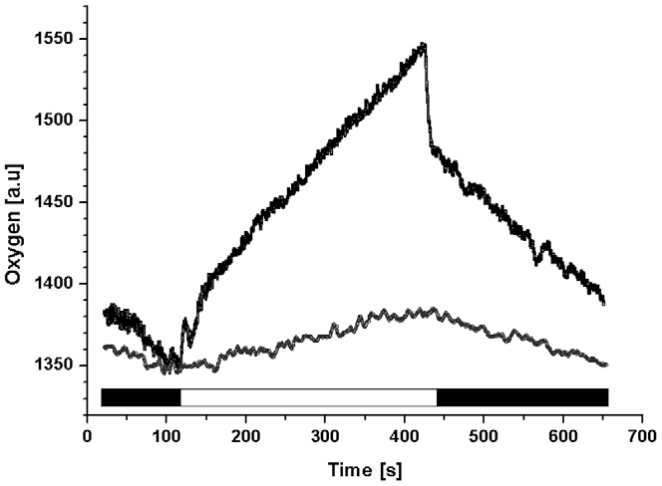
Respiratory and photosynthetic activities of *C. velia*. Representative data of low (grown under HL conditions; grey line) and high (mid-morning maximum under sinusoidal light∶dark cycle; black line) photosynthetic rates are shown. Oxygen concentration was continuously monitored using Clark-type electrode in the dark (black banner) or in the light (white banner). For better comparison, curves were artificially shifted to the same initial value at the time 115 s. Data were normalized to chl *a* concentration of samples.

An alternative possibility explaining high carbon fixation rates in high oxygen concentrations could be the occurrence of some alterative, oxygen consuming, electron flow, e.g. Mehler reaction or chlororespiration. During the Mehler reaction, the O_2_ produced by PSII is reduced again by PSI thereby decreasing its concentration [66]. Chlororespiration is defined as a respiratory-like electron transport activity from NAD(P)H to O_2_, catalysed by NADH dehydrogenase and the plastid-localized terminal oxidase (PTOX) enzyme [58], [67]. Even though PTOX has not yet been found in dinoflagellates nor their closest relatives (ciliates, perkinsea and apicomplexa; see Peltier et al. [68]), constitutive chlororespiration has already been proposed for a HL acclimated clade A *Symbiodinium* [69]. These alternative electron sinks would not only reduce the O_2_ concentrations in chloroplast, but also generate large trans-thylakoid ΔpH, provide the extra ATP and enhance photoprotection by NPQ see [70] for review. We did observe an uncoupling of ETR_PSII_ from the rate of gross oxygen production by PSII from the midday until late afternoon (see Fig. 4A) suggesting the presence of some alternative electron flow to oxygen. Moreover, we have observed a temporary fast post-illumination decrease in O_2_ concentration (Fig. 5) at the time of maximal oxygen evolution that may play a role in oxygen consumption. Interestingly, this respiration occurs only for sinusoidal grown cells with maximal rate of CO_2_ fixation and was absent in HL and LL grown cells. We hypothesize that the observed respiration may represent a mechanism by which the O_2_ concentration is reduced and thus the oxygenase activity of RuBisCO type II is minimized so that RuBisCO is turned towards higher carboxylation. This oxygen consuming process could play the role of an “optimizer” reducing O_2_ concentrations at RuBisCO. To resolve the origin of this respiration, more experiments are necessary that will also show if it proceeds in the whole thylakoid or if it is present only in some parts of membrane, where RuBisCO is located.

## Conclusions

*C. velia* is effectively a mixture of different organisms: heme synthesis as observed in Apicomplexans, simple pigmentation as in Eustigmatophyceae, primitive type II RuBisCO as in Dinoflagellates, and antenna organized as observed in Bacillariophyceae (diatoms). We have shown for the first time that this simple photosynthetic system is surprisingly efficient in photosynthetic carbon assimilation. Uniquely to *C. velia*, we propose that members of this new family of Chromeraceae use photorespiration, together with the thermal energy dissipation via NPQ, as a mechanism in their photoacclimation strategy. We propose that future studies examine the role of photorespiration in this apicomplexan. Rather than considering it a wasteful processes (compared to photosynthesis due to its high consumption of NADPH and ATP), it should be considered as mechanism for energy dissipation as well as producer of additional glycolate to its host. If photorespiration is acting in photoprotection, then corals which harbor *C. velia* (and symbiotic dinoflagellates such as *Symbiodinium*) may benefit from this particular symbiont(s).
